# FaReWell Depression – a randomized controlled trial of a physiotherapeutic program for the facial rehabilitation of wellbeing in depression

**DOI:** 10.3389/fpsyt.2026.1798454

**Published:** 2026-05-25

**Authors:** M. Axel Wollmer, Hannah Lemke, Patricia Waldvogel, Insa Neumann, Kayleigh Keller, Veronika Nölle, Nathalie Dittmer, Maksim Lucic, Barbara Walss, Tillmann H.C. Krüger, Josef Hättenschwiler

**Affiliations:** 1 Asklepios Clinic North - Ochsenzoll, Medical Faculty, Semmelweis University, Hamburg, Germany; 2 Centre for Anxiety and Depression Treatment Zurich ZADZ, Zurich, Switzerland; 3 Center for Systems Neuroscience, Hannover, Germany; 4 Department of Psychiatry, Social Psychiatry and Psychotherapy, Division of Clinical Psychology and Sexual Medicine, Hannover Medical School, Hannover, Germany

**Keywords:** embodiment, emotional proprioception, facial feedback, major depression, mind-body-medicine

## Abstract

**Introduction:**

Facial feedback mechanisms can modulate affective states. A series of clinical trials have shown that injection of botulinum toxin A can improve the symptoms of depression, probably via the disruption of proprioceptive afferences from the glabellar region to the brain. In extension of this research, we developed a physiotherapeutic program for the facial rehabilitation of wellbeing in depression (FaReWell Depression) with the objective to relax key facial muscles involved in the expression and experience of negative emotions and to tone and strengthen key facial muscles for the expression and experience of positive emotions. The program comprises a massage module with a focus on the glabellar (corrugator supercilii and procerus muscles) and perioral (mentalis and depressor anguli oris muscles) regions and a strength-training module with a focus on the cheek region (zygomaticus and orbicularis oculi muscles). We tested the therapeutic potential of this program in a randomized controlled trial.

**Methods:**

We assigned 49 patients with mild to moderate depression to either do a daily 15 minutes training session with the FaReWell Depression program or a rest exercise for the same duration as a control condition. The primary endpoint of the study was the change on the Montgomery-Åsberg Depression Rating Scale (MADRS) from the baseline to the point in time six weeks later.

**Results:**

The repeated measures analysis of covariance (ANCOVA) of the MADRS scores showed a significant group × time interaction effect (*F* = 6.37, *p* = .017, *partial η²* = .166), indicating lower depression severity in the intervention group (mean_pre_ = 20.64, SD_pre_ = 5.56, mean_post_ = 13.29, SD_post_ = 7.76) compared to the control group (mean_pre_ = 20.10, SD_pre_ = 7.23, mean_post_ = 18.26, SD_post_ = 6.78) after the training. Secondary outcome measures of among others anhedonia, short-term mood changes, facial self-perception, and facial appearance did not show significant group differences over time.

**Discussion:**

These findings provide preliminary evidence that a self-applied facial physiotherapeutic intervention can improve the symptoms of depression and warrants further investigation into this novel treatment approach.

## 1 Introduction

Our facial musculature does not only express emotions, it can also maintain, reinforce or even evoke them via proprioceptive feedback to the brain. Charles Darwin and William James described this reciprocity in the facial feedback hypothesis and meanwhile, there is a large body of experimental evidence in support of this concept (1). Facial feedback effects tend to be small and heterogeneous across experimental studies (2). However, a huge international study by The Many Smiles Collaboration involving 19 sites and almost 4000 participants has confirmed the validity of the facial feedback hypothesis (3). The corrugator supercilii muscles in the glabellar region are key muscles for the expression and, in this spirit, the experience of emotions with a negative valence. Depression involves an excess in negative emotions like sadness or anxiety and, accordingly, patients with depression exhibit a relative over-activity of these muscles (4, 5).

James’s aphorism “refuse to express a passion and it dies” represents a formula for a novel therapeutic approach in the treatment of depression. In fact, a series of randomized controlled clinical trials and meta-analyses have shown that paralyzing the corrugator supercilii and procerus muscles using a one-time injection of botulinum toxin A can rapidly, significantly, and sustainably alleviate the symptoms of depression (6–9). It is likely that the interruption of proprioceptive afferences from the face to the brain, accounts at least in part for the antidepressant effect of the treatment (10). Thus, this research validates modulation of the facial feedback loop as a therapeutic principle in the treatment of depression and opens a host of possibilities for its application.

Relaxation of glabellar and other facial muscles as well as the corresponding reduction of negative affective states may also be accomplished by non-pharmacological means as well: In a small trial, daily practice of face yoga led to measurable structural and functional changes of the face including reduced tone and reduced stiffness/increased elasticity of the corrugator supercilii muscles within eight weeks (11). In a study with 32 women, a 45-minute cosmetic facial massage had an immediate reducing effect on psychological distress (12). Moreover, in a case series with eight patients suffering from chronic facial pain, repeated application of an individualized orofacial manual therapy program reduced not only pain intensity, but also depressive symptoms within a treatment duration of four weeks (13).

Complementarily, exercise of facial muscles involved in smiling may strengthen these muscles and facilitate or reinforce positive affective states. Regular execution of a 30-minute facial training program comprising exercises for the zygomaticus muscles can significantly enhance upper and lower cheek fullness in middle-aged women within 20 weeks, probably corresponding to hypertrophy of the musculature in this area (14). These findings are confirmed by a study with 50 women, in which facial muscle exercises using a so-called Pao device for 30 seconds twice a day for 8 weeks significantly increased the cross-sectional areas of the zygomaticus muscles (15). In a prospective study with 20 postpartum women, 30 minutes of facial muscle training once a week for four weeks significantly reduced the scores on the Edinburgh Postnatal Depression Scale within the physiological range. Moreover, the training led to a happier facial expression and immediately induced a more positive mood (16).

Facial palsy specifically affecting the ability to smile is associated with an increased risk for depression (17). In a randomized controlled trial, orofacial therapy of stroke patients with a central facial palsy improved the ability to shorten the distance between the corner of the mouth and the earlobe, i.e. to smile. This correlated with an improvement in depression scores (18). In a controlled trial with 70 patients suffering from facial palsy, a 20-minute facial muscle exercise program performed six times in two weeks improved facial muscle function and reduced depression (19). A systematic review concludes that facial muscles exercise has a potential to improve depressive symptoms, elevate mood, and reduce chronic stress levels (20). However, trials investigating facial muscle exercises as a therapeutic intervention in patients with a primary depressive disorder are missing.

In extension of this research, we developed a physiotherapeutic facial training program for the treatment and rehabilitation of depression and tested it in a clinical trial. We conducted this trial under the hypothesis that patients with depression can acquire this program and carry it out on a regular basis. Moreover, we hypothesized that regular execution of its exercises can reduce the symptoms of depression and facilitate the rehabilitation of a euthymic state.

## 2 Materials and methods

### 2.1 Preliminary pilot study

Prior to the randomized controlled trial (RCT), a prototypic version of the FaReWell Depression program was tested with a small sample of healthy psychology master’s students (*n* = 4) at the University of Basel to assess its feasibility and potential effects on emotional well-being. The ethics committee of the Faculty for Psychology approved the pilot trial. After a four-week practicing period, participants demonstrated a significant increase in positive affect, as measured by the Positive and Negative Affect Schedule (PANAS; *M_pre_* = 29.00, *SD* = 3.46; *M_post_* = 35.00, *SD* = 1.41; *t*(3) = 3.57; *p* = .038) (21). This suggests that the program may elevate mood in the absence of a depressive disorder. Experience and findings from this preliminary study informed the final version of the training program and the design of the subsequent RCT in patients with depression.

### 2.2 Study design

In this proof-of-concept trial, we tested the usability and the therapeutic potential of a novel physiotherapeutic facial training program FaReWell Depression as an adjunctive intervention to reduce depressive symptoms and improve emotional well-being in individuals with mild to moderate unipolar depression. The single-blind RCT study had a delayed-start design warranting all participants access to the intervention. We conducted this study at the Centre for Anxiety and Depression Treatment Zurich ZADZ in Zurich, Switzerland between 07/2019 – 12/2024 with an interruption during the COVID-19 pandemic. The study duration per patient was 12 weeks, consisting of two phases. During the first 6 weeks, one group of participants conducted the training (intervention group, IG), while the other group completed a control routine (control group, CG). At the primary endpoint after 6 weeks, the CG also received the training and both groups continued the FaReWell Depression program for another six weeks. The study comprised five visits in total, assessing psychometric measures and adverse effects every three weeks (for detailed study schedule see **Supplementary Figure 1**). The study fulfilled the requirements of the latest version of the Declaration of Helsinki. The Ethics Committee of the Canton of Zurich granted approval for the study (BASEC-No. 2019-00076) and the trial was preregistered at clinicaltrials.gov (NCT03983291). Prior to study enrollment, all participants provided written informed consent. Participation in the study was voluntary and without monetary compensation.

### 2.3 Participants

**Figure 1:**
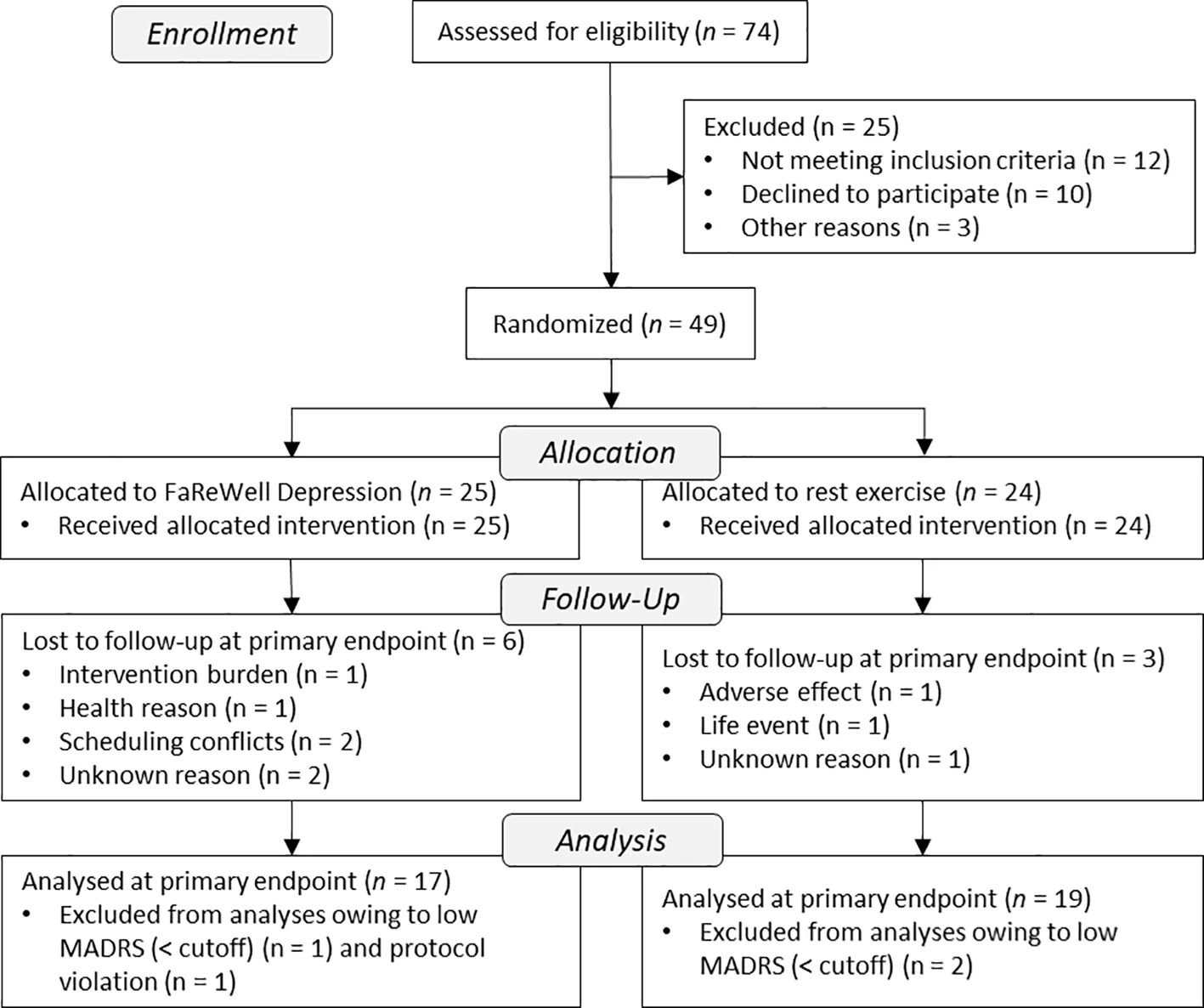
CONSORT diagram of the study procedure.

All participants were patients of the ZADZ. Either the treating psychiatrist or psychotherapists preselected patients with a high prior probability to match the requirements and referred them to the study or they became aware of it through flyers in the waiting room. Inclusion criteria were current mild-to-moderate depressive episode as defined by International Classification of Diseases (ICD-10; included diagnoses F32.0, F32.1, F33.0, F33.1), age 18–65 years, fluent command of German, and stable psychopharmacological and psychotherapeutic treatment ≥ 6 weeks before study entry, which was supposed to stay unchanged until the primary endpoint. Exclusion criteria comprised organic mental disorders (F0x), mental and behavioral disorders due to psychoactive substance use (F1x), schizophrenia, schizotypal and delusional disorders (F2x), other clinically significant psychiatric comorbidities, prior cosmetic facial procedures (e.g., lifting, botulinum toxin, fillers), as well as clinically relevant motor or sensory impairments of the face and hands, and facial dermatoses (e.g. eczema, rosacea, acne). We screened potential participants for study eligibility (visit 0) and rechecked inclusion and exclusion criteria at the baseline (visit 1). A CONSORT diagram shows the participant flow through the study (**Figure 1**). The originally targeted enrollment of 40 patients was extended to 49 to compensate for a dropout rate that exceeded our expectations of 15-20%. The sample size was informed by our previous studies on the use of botulinum toxin as a treatment for depression, which have demonstrated significant effects in comparable patient populations, presumably via similar mechanisms (6, 8).

### 2.4 Procedure and intervention (FaReWell Depression)

**Figure 2:**
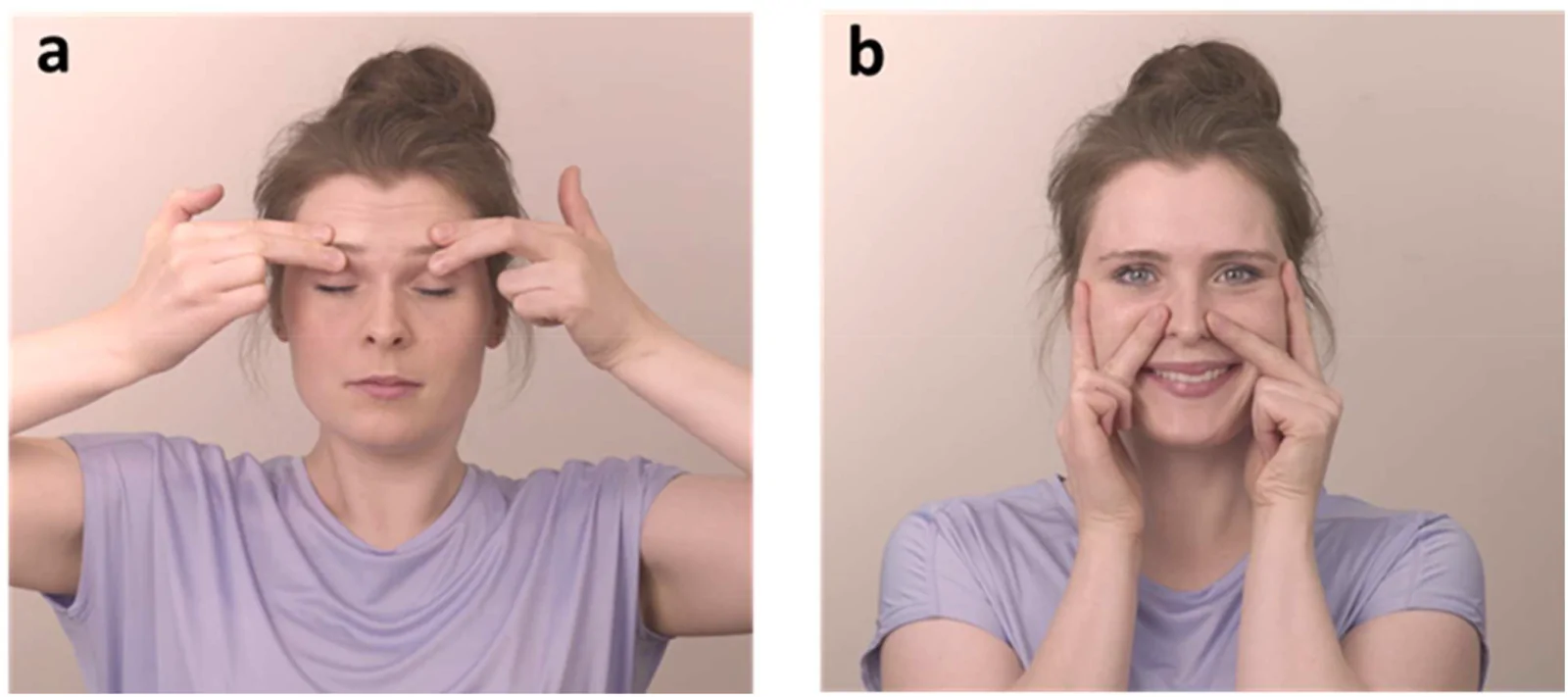
Relaxing and activating exercises from the FaReWell Depression program. Two examples: Targeted self-massage was used to relax facial muscles associated with negative emotions [here: corrugator supercilii muscles, **(a)**]. Repeated contractions against finger-applied resistance serve to activate and strengthen facial muscles associated with positive emotionality [orbicularis oculi and zygomaticus major muscles, **(b)**].

The IG received a 60-minute instruction session introducing the physiotherapeutic facial training program FaReWell Depression at the baseline (visit 1). FaReWell Depression stands for facial rehabilitation of wellbeing in depression. The program is a compilation of self-developed exercises and modified versions of exercises described in various facial programs from the wellness and beauty segment (22–26). The training comprises two modules: 1) A passive facial relaxation through self-massage first of the whole face and then targeting muscles linked to negative affect, i.e. the glabella complex with the corrugator supercilii and the procerus muscles as well as the perioral region with the depressor anguli oris and mentalis muscles, and 2) an active facial muscle strength-training involving three cyles of contractions of muscles linked to positive affect, i.e. the zygomaticus and orbicularis oculi muscles, against resistance generated by the fingers (10 repetitions per cycle) and a forced smile (held for 20 seconds). All exercises are carried out from a “half smiling”, as known from Dialectic Behavioral Therapy, basic position and each training session is concluded with a minute of tapping and stroking the face (27). The duration of a training session was about 15 minutes. Examples of two exercises are provided in **Figure 2**. The physiotherapist explained, demonstrated and trained the different exercises with all participants individually. To support home practice, participants received a printed manual with series of images and descriptions of the exercises as well as access to a professionally produced, internet-based video-guide (@michaelismedia; see **Supplementary Data 1** for description of the individual exercises). We instructed participants to practice a training session at home every day for the following six weeks. To ensure correct execution, the physiotherapist checked participants’ performance after one week and provided corrections if necessary. The same physiotherapist conducted both the initial instruction of the intervention at the baseline and the subsequent assessment of correct exercise performance after one week. To facilitate and to assess adherence, we asked the participants to document the completion of the exercises in a study diary. If desired, they received a daily reminder via SMS provided by LOX24 (https://www.lox24.eu/). The CG received a brief introduction to a non-specific daily 15-minute rest exercise. We instructed them to find a quiet place where they would not be disturbed or interrupted, to sit in a comfortable, upright position, to close their eyes (optional), and to breathe calmly and evenly.

### 2.5 Randomization and blinding

Upon study enrollment, patients were randomly assigned to either the intervention or the control condition in a 1:1 ratio using block randomization provided by sealed envelope™ (https://www.sealedenvelope.com/simple-randomiser/v1/lists). Patients were informed of their allocation at the baseline visit. Given the nature of the intervention, blinding of patients and the instructing physiotherapist was not feasible. However, all study staff involved in psychometric rating remained blinded to individual group allocation, resulting in a single-blind study design. Study staff were instructed not to ask about patients’ group allocation, and patients were instructed not to reveal their allocation during study visits.

### 2.6 Measures

We assessed depressive symptoms using both clinician-rated and self-reported measures. Clinically trained raters administered the Montgomery–Åsberg Depression Rating Scale (MADRS) to evaluate the severity of current depressive symptoms (28). To ensure consistency in clinical ratings, the raters underwent an internal training in the use of the MADRS and, whenever possible, the same clinician conducted the assessments across all visits for each participant. Response was defined as a 50% reduction from the baseline score. Remission was defined as MADRS score of <10, in line with a previous recommendation (29). In addition, all patients completed the Patient Health Questionnaire-9 (PHQ-9) as a brief self-report measure of current depressive symptom severity (30) and the Snaith–Hamilton Pleasure Scale (SHAPS-D) to assess anhedonia (31). Moreover, the “Fragebogen zu Leistungsfähigkeit und Wohlbefinden” was used to measure functioning and well-being as an overall score (in %). In addition, we evaluated the three subscales (domains: work, social life and private life/family) and a sum scores of the three domains (Swiss Group Project: “Depression: Complete Remission/Well-Being, see **Supplementary Data 2** for translation of the scale). All instruments were administered at the baseline (visit 1), at the primary endpoint (week 6, visit 3) and at the end of the study (week 12, visit 5). For better temporal resolution in the documentation of depressive symptoms, we also collected MADRS and PHQ-9 data after three weeks (visit 2) and after nine weeks (visit 4). Furthermore, patients completed the Multidimensional Mood State Questionnaire (MDBF) at home every two weeks on a day of the participant’s choice, immediately before and after completing a training session (32). The MDBF has three subscales (mood, alertness and calmness) and was used to capture short-term effects of the intervention. Moreover, we queried treatment stability, problems with the execution of the study intervention, and side effects at every study visit. We asked participants whether they felt any tension or paresthesia in the facial area, which we documented on a facial map. To capture possible changes in facial appearance or expression, we took two standardized portrait photos of each participant, one with a neutral and one with a smiling expression, at the baseline, after six weeks, and after 12 weeks. The photos underwent pre-processing and were analyzed with the FaceReader (version 9.1.5) by Noldus Information Technology (https://www.noldus.com). FaceReader is a validated software package developed for automatic, algorithm-based analysis of facial features (e.g. identification and quantification of facial expressions, emotional states, and facial muscle activity) based on standardized images or videos. For each participant, standardized images from week 0 and week 6 were imported into the software, which automatically computed variables including facial action units (AU4, AU6, AU10, AU12, AU15, and AU17), valence, arousal, and emotion intensity (neutral, happy). These photos were also rated by 20 medical students at the Asklepios Campus Hamburg of the Semmelweis University. In brief, the photos were scrambled to generate a blinded random order. Each photo was presented on a screen for 60 seconds and the students were asked to rate the affective valence of the facial expression on a -10 (very negative) to 10 (very positive) and the apparent facial tension on a 0 (completely relaxed) to 10 (very tense) Likert scale using a digital questionnaire.

### 2.7 Data analysis

We performed all statistical analyses in IBM SPSS Statistics (version 29). We evaluated the study including all participants with MADRS data at visits 1 and 3 and without relevant protocol violations (per-protocol analysis). All statistical analyses were adjusted for age and sex. All tests were performed two-tailed with a significance level of α <.05.

#### 2.7.1 Primary endpoint analysis

The predefined primary efficacy analyses examined antidepressant effects of the intervention after six weeks in participants with MADRS data available at visits 1 and 3. A 2×2 repeated-measures ANCOVA with group (IG, CG) as between-subjects factor and time (week 0, week 6) as within-subjects factor was conducted using MADRS scores as dependent variable, testing group-by-time interaction effects.

Additionally, we calculated response and remission rates at the primary endpoint. Participants achieving ≥50% reduction in MADRS from baseline (week 0) to the primary endpoint (week 6) were classified as responders and participants with MADRS scores <10 at the primary endpoint were classified as remitters. We compared proportions of responders and remitters between IG and CG using Chi-square tests.

#### 2.7.2 Secondary endpoint analyses

The following secondary analyses examining both short-term and longer-term effects of the intervention on multiple symptom dimensions were performed.

A. Self-reported changes in symptoms and well-being: Effects of the intervention on self-reported depression, anhedonia and functioning and well-being were analyzed using the same 2×2-ANCOVA as used in the primary analyses with PHQ-9, SHAPS-D and “Fragebogen zu Leistungsfähigkeit and Wohlbefinden” as dependent variables.

B. Longer-term intervention effects: Sustained effects of the intervention were assessed using 3×1-ANCOVAs with time (week 0, week 6, week 12) as within-subjects factor and MADRS, SHAPS-D, and PHQ-9 as dependent variables, in the IG exclusively. To further examine longer-term intervention effects within the IG, prespecified pairwise comparisons were conducted to assess changes from baseline (week 0) to post-intervention (week 6) and the maintenance of effects at follow-up (week 12).

C. Immediate training-related changes: Short-term changes in mood and well-being directly before and after the training sessions were analyzed using 2×2-ANCOVAs with group (IG, CG) as between-subjects factor and time (pre-session, post-session) as within-subjects factor. The dependent variables were MDBF scores of the three subscales ‘calmness’, ‘wakefulness’ and ‘mood’, averaged across weeks 2, 4 and 6.

D. Effects on facial features: Intervention-related effects on objective facial features and action units were examined using 2×2-ANCOVAs for FaceReader outcomes, including AU4, AU6, AU10, AU12, AU15, and AU17, emotion intensity, valence, and arousal as dependent variables. Time (week 0, week 6) served as the within-subjects and group (IG, CG) as the between-subjects factor. In addition, 2×2-ANCOVAs were conducted using the students’ evaluations, again with group as a between-subjects factor and time as a within-subjects factor. Dependent variables were the ratings of the affective valence of the facial expression and the perceived tension of the face.

E. Effect sizes and adherence: To calculate effect sizes of the intervention, paired-sample t-tests were conducted for pre- to post-training changes in MADRS, PHQ-9 and SHAPS-D scores (week 0–6 in the IG; week 6–12 in the CG). Additionally, in the IG, ΔMADRS scores were calculated as post- minus pre-training values and used to examine whether adherence rate was correlated with antidepressant effects (change in MADRS scores) using Pearson’s correlation.

F. Control analysis: In case of significant primary endpoint effects and to examine whether the intervention had similar antidepressant effects in the IG and CG, ΔMADRS scores (week 0–6 in the IG; week 6–12 in the CG) were used to calculate independent-sample t-test, serving as a control analysis with no significant group differences expected.

## 3 Results

### 3.1 Descriptive and clinical characteristics

The IG and CG did not differ significantly regarding demographic (age, sex, years of education) and clinical (comorbid psychiatric disorders, psychotropic medication, number of depressive episodes or history of suicide attempts) baseline variables (**Table 1**). Three Participants were excluded from the trial, because they were not depressed at the baseline (MADRS < 10). In one participant in the IG there was a major change in the concomitant treatment (new antidepressant medication with sertraline), which we rated as a protocol violation and consequently excluded the participant from the analyses. There were nine dropouts in the whole cohort, six in the IG and three in the CG. The dropouts did not differ significantly from the remaining sample in the baseline MADRS scores (MADRS_dropouts_ = 20.56, MADRS_totalsample_ = 20.36, *p* = .939) and all other demographic and clinical baseline variables except for a marginal difference in years of education (*p* = .049; see **Supplementary Table 1**). The final sample consisted of 17 patients in the IG and 19 participants in the CG. The adherence rate in this sample, as documented in the patient diaries, was similar between the IG and the CG (73.52% vs. 79.69%, **Table 2**). The intervention and the control condition were both well tolerated. Only few participants reported facial tension or paresthesia at the baseline or during the further course of the study (see **Supplementary Table 2**).

**Table 1:** Demographic and clinical characteristics of the study sample.

Characteristics	Intervention group (IG) (n = 17)	Control group (CG) (n = 19)	*P*-value
	Mean (s.d.)	Mean (s.d.)	
*Sociodemographic characteristics*			
Age (years)	43.18 (13.19)	42.84 (12.60)	.938^1^
Sex (female/male)	13/4	13/6	.590^2^
Years of Education	13.76 (2.22)	14.72 (2.21) (n = 18)	.211^1^
*Clinical characteristics*			
Number of depressive episodes	5.18 (4.70) (n = 16)	2.71 (2.05) (n = 17)	.056^1^
Number of suicide attempts	.117 (.33)	.158 (.50)	.781^1^
*Lifetime psychiatric comorbidity (yes/no)*	5/12	7/12	.637^2^
Anxiety disorders	2	4	–
Eating disorders	2	0	–
ADHD	1	1	–
Others	0	2	–
*Psychotherapeutic Treatment (yes/no)*	15/2	15/4	.455^2^
*Medical treatment at baseline (yes/no)*	12/5	8/11	.086^2^
Mono therapy	9	7	–
Combined therapy	2	1	–
Augmentation therapy	1	0	–

^1^Two-sample t-tests, ^2^Chi-squared t-test.

IG, Intervention Group; CG, Control Group; ADHD, Attention-Deficit-Hyperactivity Disorder.

**Table 2:** Outcome variables of the study sample.

Variables	Intervention group (IG) (n = 17)	Control group (CG) (n = 19)	
	Mean (s.d.)	Mean (s.d.)	*P*-value
Primary outcome variables			
Adherence rate (in %)	73.52 (15.15)	79.69 (14.36)	.218^1^
MADRS week 0	20.64 (5.56)	20.10 (7.23)	.804^1^
MADRS week 3	16.63 (7.50) (n = 16)	16.27 (6.69) (n = 18)	.890^1^
MADRS week 6	**13.29 (7.76)**	**18.26 (6.78)**	**.024^1^**
MADRS week 9	15.31 (8.00) (n = 13)	13.28 (6.33) (n = 18)	.437^1^
MADRS week 12	14.78 (7.76) (n = 14)	13.25 (6.86) (n = 16)	.570^1^
ΔMADRS (week 6 – week 0)	**-7.35 (5.75)**	**-1.84 (7.27)**	**.009^1^**
Responders *yes/no (%)*	6/11 (35.29%)	2/17 (10.52%)	.074^2^
Remission *yes/no (%)*	5/12 (29.41%)	2/17 (10.52%)	.163^2^
Secondary outcome variables			
*Self-reported depression*			
PHQ-9 week 0	11.70 (5.31)	11.37 (3.66)	.824^1^
PHQ-9 week 3	8.76 (4.30)	8.61 (4.25) (n = 18)	.916^1^
PHQ-9 week 6	8.23 (5.42)	10.16 (3.90)	.227^1^
PHQ-9 week 9	9.23 (4.98) (n = 13)	8.11 (4.24) (n = 18)	.506^1^
PHQ-9 week 12	7.71 (3.36) (n = 14)	9.11 (5.19) (n = 17)	.321^1^
*Self-reported Anhedonia*			
SHAPS-D week 0	1.88 (2.09)	2.89 (3.31)	.287^1^
SHAPS-D week 6	1.47 (2.35)	2.39 (3.15) (n = 18)	.334^1^
SHAPS-D week 12	1.35 (1.90) (n = 14)	1.29 (1.72) (n = 17)	.924^1^
*Self-reported functioning and well-being (F+WB)*			
Percentage of F+WB week 0 (in %)	52.06 (17.14)	52.28 (16.12) (n = 18)	.969^1^
Percentage of F+WB week 6 (in %)	64.41 (22.90)	57.06 (20.77) (n = 17)	.334^1^
Percentage of F+WB week 12 (in %)	59.42(26.28)(n = 14)	59.38 (20.40) (n = 16)	.995^1^
Sum of F+WB week 0 (total score)	49.40 (18.46) (15)	49.17 (14.11) (18)	.967^1^
Sum of F+WB week 6 (total score)	55.84 (16.39) (13)	46.37 (16.37) (16)	.133^1^
Sum of F+WB week 12 (total score)	55.67 (14.68) (12)	51.58 (11.48) (12)	.456^1^
Domain: Work week 0	4.96 (2.24) (n = 15)	5.12 (1.73) (n = 18)	.814^1^
Domain: Work week 6	5.84 (2.42) (n = 13)	5.06 (2.48) (n = 16)	.401^1^
Domain: Work week 12	**6.38 (2.16) (n = 13)**	**4.69 (1.90) (n = 13)**	**.022^1^**
Domain: Social life week 0	5.11 (2.81)	5.52 (1.96)	.613^1^
Domain: Social life week 6	5.68 (2.43) (n = 15)	5.09 (1.97) (n = 17)	.455^1^
Domain: Social life week 12	5.89 (2.50) (n = 12)	5.33 (1.89) (n = 16)	.509^1^
Domain: Personal life/Family week 0	5.70 (2.38)	5.91 (2.37)	.797^1^
Domain: Personal life/Family week 6	6.22 (2.36) (n = 15)	5.11 (1.91) (n = 17)	.155^1^
Domain: Personal life/Family week 12	6.58 (1.97) (n = 12)	6.48 (1.69) (n = 15)	.895^1^
*Multidimensional Mood State Questionnaire (MDBF)*			
Subscale: calmness (pre-training)	24.18 (5.39)	22.27 (4.03)	.235^1^
Subscale: calmness (post-training)	27.56 (4.97) (16)	26.46 (3.94)	.471^1^
Subscale: wakefulness (pre-training)	20.06 (3.98)	21.64 (4.29)	.261^1^
Subscale: wakefulness (post-training)	23.06 (5.21) (16)	23.29 (4.12)	.887^1^
Subscale: mood (pre-training)	25.22 (5.43)	25.06 (3.63)	.920^1^
Subscale: mood (post-training)	27.56 (4.97) (16)	26.46 (3.94)	.556^1^

Significant group differences are highlighted in bold. ^1^Two-sample t-tests, ^2^Chi-squared t-test.

IG, Intervention Group; CG, Control Group; MADRS, Montgomery–Åsberg Depression Rating Scale; PHQ-9, Patient Health Questionnaire–9; SHAPS-D, Snaith–Hamilton Pleasure Scale; F+WB, functioning and well-being; Responders were defined as change in MADRS scores of ≥50% from pre training to the primary endpoint, Remission was defined as a symptom reduction in MADRS scores to <10 at the primary endpoint.

### 3.2 Primary endpoint analysis

**Figure 3:**
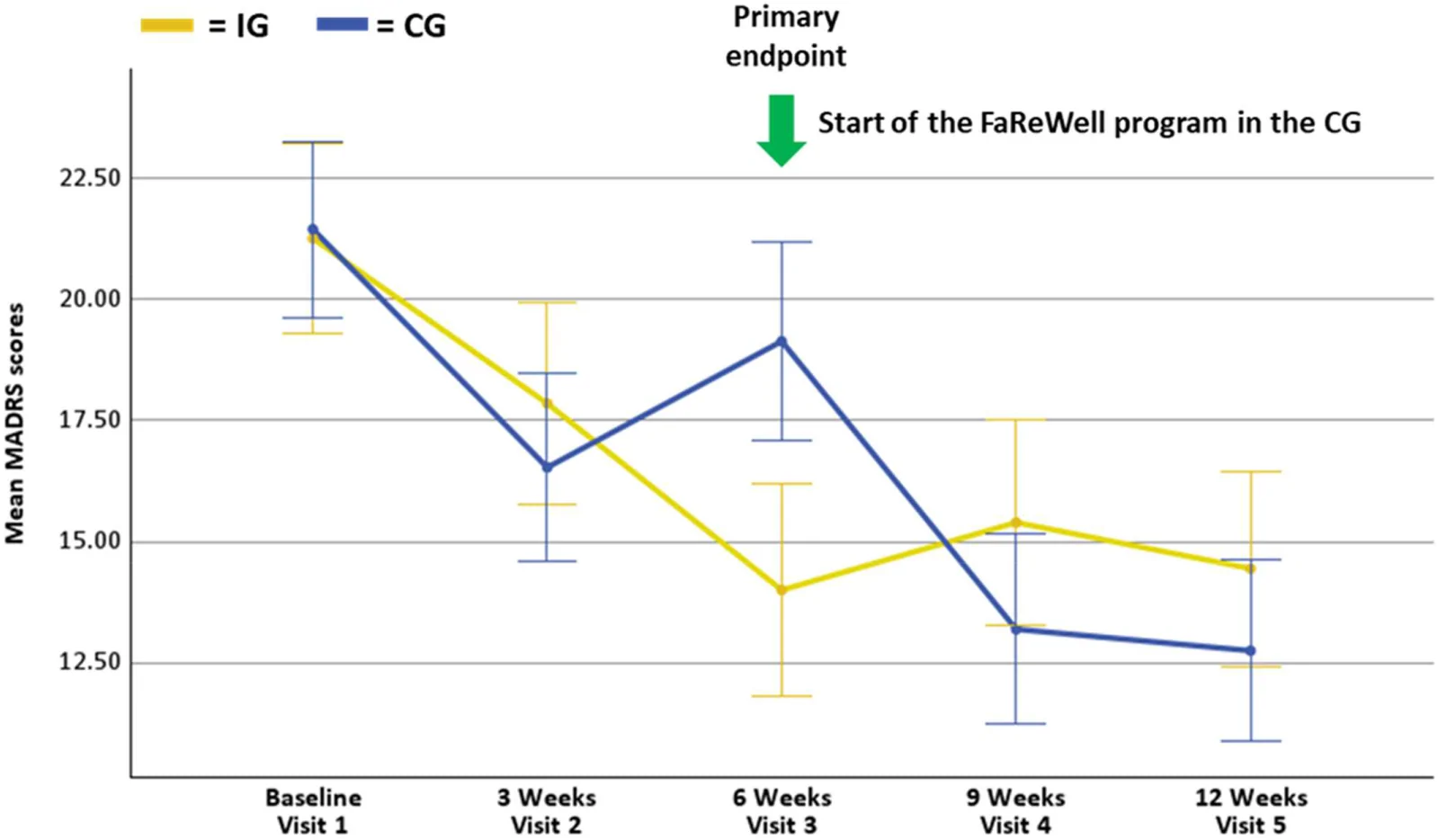
Group × time interaction in mean MADRS scores. Within the first three weeks, there was a similar MADRS score reduction in both groups. In the next three weeks, the control group (CG) showed a mean score resurgence while in the intervention group (IG) the mean score continued to decline linearly. At the primary endpoint, there was a significant improvement in the symptoms of depression in the IG as indicated by the group (IG vs. CG) × time (six weeks vs. baseline) interaction for MADRS scores with *F*_(1,32)_ = 6.37, *p* = .017, *partial η²* = .166. A *post-hoc* t-test for group difference in MADRS scores after 6 weeks confirmed superiority of the FaReWell Depression program over the control condition with *t*_(34)_ = 2.05, *p* = .024. The IG remained largely stable on this level for the next six weeks until the end of the study. After switching to the FaReWell Depression program (green arrow), MADRS scores in the CG dropped steeply in the next three weeks and then remained stable until the end of the study. Both groups attained a similar improvement in the symptoms of depression after six weeks on the FaReWell Depression program as indicated by an independent-samples t-test for ΔMADRS scores at this time point (*p* = .654). Error bars represent standard errors.

The primary analysis revealed a significant group × time interaction for MADRS scores with *F*_(1,32)_ = 6.37, *p* = .017, *partial η²* = .166 (**Figure 3**). *Post-hoc* t-tests confirmed a significant difference at week 6 between the groups, with lower MADRS scores in the IG compared to the CG *t*_(34)_ = 2.05, *p* = .024, *Cohen’s d* = 0.68. To test the robustness of the effect, we repeated the ANCOVA including the participant who had originally been excluded due to the protocol violation (see above). The group × time interaction remained statistically significant (*F*_(1,33)_ = 5.08, *p* = .031, *partial η²* = .133).

About 35.29% of the IG responded to the intervention, while the response rate in the CG was 10.52%. However, the χ²-test yielded only a trend for a group difference in response rate at the primary endpoint (*p* = .074). Following the intervention, 29.41% of patients in the IG achieved remission (corresponding to a MADRS score <10 at the primary endpoint), whereas remission rate in the CG was 10.52%, yielding no significant group difference (*p* = .163). Descriptive statistics of outcome variables are provided in **Table 2**.

### 3.3 Secondary endpoint analyses

A. Self-reported changes in symptoms and well-being: The 2×2 ANCOVAs on self-reported functioning and well-being showed no significant group × time interactions for the overall score (*p* = .196), the subscale work *(p* = .176), subscale social life (*p* = .089) and subscale personal/family life (*p* = .083). The 2×2 ANCOVAs also showed no significant main or interaction effect over time for self-reported symptoms of depression (PHQ-9; all *p*>.241) and anhedonia (SHAPS-D; all *p*>.755).

B. Longer-term intervention effects: The 3×1 ANCOVAs across weeks 0, 6 and 12 in the IG showed no significant main effect of time for MADRS (*p* = .783) or PHQ-9 scores (*p* = .458). A significant main effect of time emerged for SHAPS-D scores with *F*_(2,22)_ = 4.07, *p* = .033, *partial η²* = .267. *Post-hoc* paired-sample t-tests did not reveal any significant differences between time points. For MADRS scores, the prespecified pairwise comparisons revealed a significant reduction in symptom severity from baseline (week 0) to post-intervention (week 6) (*t*_(16)_ = 5.28, *p* <.001, *Cohen’s d* = 1.28), but not for the comparison from week 6 to week 12 (p = 769), i.e. the score reduction remained stable over time in the IG. For the PHQ-9 and the SHAPS-D, no significant pairwise comparisons emerged (all *p*>.062).

C. Immediate training-related changes: For the MDBF subscales “mood”, “wakefulness”, and “calmness” no significant group × time interactions were observed (all *p*>.083). Only for the subscale “calmness” a main effect of time emerged with *F*_(1,31)_ = 11.57, *p* = .002, *partial η²* = .272, indicating a significant increase in calmness from pre to post training in both groups.

D. Effects on facial features: The analyses of intervention effects on facial features as measured by the FaceReader revealed neither significant group × time interaction effects nor main effects of group or time for any of the analyzed facial AUs, emotion intensity (neutral, happy), valence, or arousal (all *p*>.103, data not shown). Analyses of intervention effects on facial features, as evaluated by the students, did not reveal any significant group × time effects either (all *p*>.381, data not shown).

E. Effect sizes and adherence: The paired-sample t-tests showed significant reductions in MADRS (*t*_(33)_ = 5.28, *p* <.001, *Cohen’s d* = 0.91), SHAPS-D (*t*_(33)_ = 2.48, *p* = .018, *Cohen’s d* = 0.43) and PHQ-9 scores (*t*_(34)_ = 2.51, *p* = .017, *Cohen’s d* = 0.42) in the entire sample (IG and CG), corresponding to medium to large effect sizes of the intervention. Pearson’s correlation between training adherence (in %) and ΔMADRS in the IG did not reach significance (*p* = .808).

F. The independent-samples t-test comparing ΔMADRS scores of the six-week training between the IG and CG revealed no significant difference (*p* = .654).

## 4 Discussion

### 4.1 Feasibility and acceptability

In this proof-of-concept trial, we show that patients with mild to moderate depression manage to learn and regularly apply the FaReWell Depression program with instruction and supervision by a physiotherapist and the support of a manual and video. Although there were more dropouts in the IC than in the CG, only one patient dropped out due to the burden associated with the intervention. The adherence to the program was surprisingly good with an almost 75% achievement rate of the provided daily training sessions. Adherence was supported and documented by a training diary and study participant had the opportunity to receive daily SMS reminders to execute the exercises.

### 4.2 Effects on symptoms of depression and mood states

The FaReWell Depression program led to an improvement in the symptoms of depression at the primary endpoint of the study after six weeks of training. In the per protocol analysis of the MADRS clinician rating, the experimental condition was significantly superior to the control condition. The improvement corresponded to a large effect size and a response rate of 35%. There was no correlation between achievement rate and the improvement in the symptoms of depression, i.e. no dose-response relationship. The original control group experienced a similar symptomatic improvement when they switched to the experimental condition and the experimental group sustained the improved state until the end of the study. In the PHQ-9 self-rating, there was a numerical, but not statistically significant superiority of the experimental condition. These findings indicate that FaReWell Depression may be an actionable and effective adjunctive intervention in the treatment of depression.

There was a numerical reduction of the SHAPS-D raw values, which was more, yet not significantly more pronounced with the experimental condition. However, anhedonia scores were generally low and did not leave much room for improvement. Scores of functioning and wellbeing tended to go up in the IG and down in the CG. This indicates that FaReWell Depression may actually help to attain “rehabilitation of wellbeing in depression”, which is the eponymous purpose of the program. However, the score differences were not statistically significant.

The exercises had no immediate effects on MDBF scores, except for an unspecific increase in calmness. It is possible that potential beneficial effects were foiled by potential detrimental effects of feeling insecure or insufficient while executing the program. Overall, however, FaReWell Depression does not seem to be suitable for a short-term modulation of mood states in patients with depression. This is in contrast to previous studies that did report immediate effects of facial interventions on mood states (12, 16). In one of these studies, patients passively received a face massage from a therapist and did not apply it on themselves like in our program. Differences in the perception and the neural effects between self-touch and touch by another person may partly explain this discrepancy (33).

### 4.3 Effects on facial appearance and perception

FaReWell Depression did not change the facial muscular activation pattern at rest and during smiling as assessed on standardized photos using the FaceReader software and by a jury of 20 medical students. Hence, the reduction in the symptoms of depression does not appear to translate into an altered facial expression.

Only few participants reported paresthesia or sensation of tension in the facial area. This may indicate that the conscious proprioception or self-perception of the face is probably not systematically altered in patients with depression.

### 4.4 Limitations

The major limitations of this study are the comparably low number of participants and the impossibility of blinding the patients for group allocation. The positive primary endpoint is seen alongside a host of negative secondary endpoints. Therefore, the observed efficacy is a preliminary finding that needs replication in a larger study. In such a sufficiently powered trial, numeric trends for small beneficial effects in secondary outcome measures may be verified, too. Moreover, FaReWell Depression is a complex intervention making it difficult to disentangle the contributions of its individual components to the antidepressant effect. The massage and activating exercises aim to relax or strengthen facial muscles, respectively. However, we do not know if the program actually attained a change in the tone of the targeted muscles. We did not measure muscle tone and hardly any of the participants reported a subjective feeling of muscle tension in the targeted facial areas. We found an antidepressant effect of the program in a per protocol analysis. This probably represents a best-case scenario. Overburdening with the program or even the control condition may have led to loss of patients with more severe depression or a worse clinical course during the study. Since there was a higher dropout rate in the experimental group, this may have introduced a bias and inflated the group difference. However, the patients who dropped out did not differ significantly from those who completed the study in any of the clinical or demographic baseline variables except for a minor difference in years of education.

### 4.5 Relation to previous research on botulinum toxin injections as a treatment for depression

The FaReWell Depression program and this study were inspired by our previous research into the use of botulinum toxin A as a treatment for depression. Relying on the same concept of improving affective states via the modulation of emotional proprioception from the face, the two approaches have some important differences. While botulinum toxin injection results in a complete and prolonged paralysis of the glabellar musculature, FaReWell Depression aims at a subtle relaxation of these muscles. Unlike botulinum toxin A, FaReWell Depression activates facial muscles involved in the expression and experience of positive emotions. Thus, it has the potential to actively build up a more positive emotional state. While glabellar paralysis by injection of botulinum toxin A impairs the expression of negative emotions, FaReWell Depression does not limit facial expression. Botulinum toxin A injection is a passively received treatment that only requires a one-time commitment and is therefore suitable for patients with severe drive disorder. FaReWell Depression is a self-applied program that requires the drive for regular training. Thereby, it may convey behavioral activation and an experience of self-efficacy.

Botulinum toxin A injection is an invasive procedure that is safe and tolerable, but still has a risk for side effects like ptosis. FaReWell Depression has hardly any side effects.

### 4.6 Possible mechanisms of action

FaReWell Depression may exert its antidepressant effect through various possible mechanisms of action. We developed it based on the facial feedback hypothesis and the assumption that the relaxation of key muscles for the expression of emotions with negative valence, i.e. sadness, fear or anger, of which there is an excess in depression, will reduce the experience of theses emotions. Conversely, we supposed that the activation of key muscles for the expression of emotions with positive valence, i.e. happiness, which is lacking in depression, would promote a more positive affective state. The latter may be the most relevant component, because reinforcement and generation of feelings of happiness through activation of the smiling musculature seems to be the most robust facial feedback effect (3).

It is possible that offering an intervention with a compelling rationale raises expectations exceeding those in the control condition. Moreover, the initial instruction and supervision for the FaReWell Depression program provided more attention and contact from the study staff than the instruction for the control condition, which may have contributed to the better outcome in the intervention group. This may be assimilated in future confirmatory trials. Because blinding the patients to treatment allocation was not possible, differential placebo effects may contribute to or account for the outcome differences between the groups (34). However, the initial symptom reduction observed in both groups may represent similar placebo effects in both groups.

The regular execution of the training and specifically the activation of smiling muscles represents a behavioral activation, which is a major active factor in the treatment of depression and conveys an experience of self-efficacy (35, 36).

Facial self-touches are a common behavior and may serve as a means of self-soothing and arousal reduction in distressing situations and during social interactions (33). It is possible that the facial self-touches associated with the massage and activation exercises are involved in the antidepressant effect. This may also imply the acupressure of specific meridian points that are also targeted in Emotional Freedom Techniques (EFT) (37). However, the lack of immediate positive effects in the MDBF scores argues against this possibility. In summary, specific and unspecific mechanisms may have contributed to the observed clinical improvement.

### 4.7 Therapeutic potential and future directions

FaReWell Depression is a novel mind-body-medicine (MBM) intervention that combines facial muscle exercise and massage. MBM uses the reciprocal interconnection of mental and physical processes for the treatment of mental disorders like depression and comprises both muscle relaxation and activation techniques (38).

There is some evidence for efficacy of massage therapy in depression (39, 40). Physical activity and exercise are effective both as stand-alone and adjunctive interventions in the treatment of depression and attain effect sizes comparable to those of psychotherapeutic or pharmacological interventions (41–43). It is conceivable that, along with other mechanisms of action, they may exert their antidepressant effect via changes in interoceptive and proprioceptive feedback from the body to the brain. With regard to emotional proprioception, it is noteworthy that these physical interventions largely spare the face although the proprioceptive feedback from this region has specific regulatory effects on affective states as described by the facial feedback hypothesis (10). The FaReWell Depression program may contribute to closing this gap.

Arbitrary movement of the facial action units or facial muscles that yield a smile represents a behavioral activation that counteracts negative affective states and may contribute to overcoming anhedonia and channel positive emotions (36). This is important because on the one hand anhedonia often persists even after clinical remission of depression and on the other hand, an early return of positive emotions is a good predictor of achieving remission (44, 45). A similar approach is followed by laughter-inducing therapies (46).

FaReWell Depression may not be suitable for patients with severe depression, because these patients may lack the critical amount of drive to execute the regular exercises required by the program. However, in these cases the same approach may be put into effect by passive application. As long as drive is lacking, facial massage may be applied by a therapist and activation of the smiling muscles may occur by facial neuromuscular electrical stimulation, which may result in more positive emotions and reduced symptoms of depression (12, 47–49).

In conclusion, the FaReWell Depression program is simple and compact and does not require any technical aids, so that patients with mild to moderate depression can learn it quickly and can apply it anytime and anywhere, contributing to the experience of self-efficacy. This proof-of-concept trial shows the therapeutic potential of the program as an adjunctive treatment for depression. It corroborates the facial musculature as a valid target in the treatment of depression. Larger trials are warranted to confirm the antidepressant effect of the program, to carve out its most effective components, and to further explore if it can really contribute to the rehabilitation of wellbeing in depression.

## Data Availability

The raw data supporting the conclusions of this article will be made available by the authors, without undue reservation.
