# Correction: FaReWell Depression – a randomized controlled trial of a physiotherapeutic program for the facial rehabilitation of wellbeing in depression

**DOI:** 10.3389/fpsyt.2026.1959551

**Published:** 2026-08-19

**Authors:** M. Axel Wollmer, Hannah Lemke, Patricia Waldvogel, Insa Neumann, Kayleigh Keller, Veronika Nölle, Nathalie Dittmer, Maksim Lucic, Barbara Walss, Tillmann H.C. Krüger, Josef Hättenschwiler

**Affiliations:** 1 Asklepios Clinic North - Ochsenzoll, Medical Faculty, Semmelweis University, Hamburg, Germany; 2 Centre for Anxiety and Depression Treatment Zurich ZADZ, Zurich, Switzerland; 3 Center for Systems Neuroscience, Hannover, Germany; 4 Department of Psychiatry, Social Psychiatry and Psychotherapy, Division of Clinical Psychology and Sexual Medicine, Hannover Medical School, Hannover, Germany

**Keywords:** embodiment, emotional proprioception, facial feedback, major depression, mind-body-medicine

In the **Acknowledgements**, thanks to “Renée Ambarees Isermann, Gary Sikorski and Dominique de Quervain” were erroneously omitted. The corrected statement is:

“We thank the whole staff of the ZADZ, especially Karin Yerebakan-Schaller and Nicolas Weyland, for their continuous support in the execution of this study; Kerstin Michaelis for the production of the video guide; Renée Ambarees Isermann, founder of Yoga4Face^®^, for helpful discussions and her professional perspective on facial training, aesthetics, and face embodiment; Gary Sikorski for providing his Happy Face Yoga Cards; and Dominique de Quervain for supporting the preliminary pilot study.”

The original version of this article has been updated.

