# Supplementary material for: FaReWell Depression – a randomized controlled trial of a physiotherapeutic program for the facial rehabilitation of wellbeing in depression
Source: Front Psychiatry. 2026 May 25;17:1798454. doi: 10.3389/fpsyt.2026.1798454 (PMC13243274; doi:10.3389/fpsyt.2026.1798454)
Supplement: Supplementary file 1 [file DataSheet1.pdf]

frontiers

Supplementary Material

FaReWell Depression – a Randomized Controlled Trial of a Physiotherapeutic Program for the Facial Rehabilitation of Wellbeing in Depression

1 Supplementary Data

Supplementary Data 1

Translation of the Instructions of the individual exercises of the **FaReWell Depression program** (use of google translate with subsequent editing):

Half-smile
The half-smile is the starting position from which you perform all exercises and to which you return after each exercise.
Just pull the corners of your mouth very slightly to the side and upwards.
Keep your lips relaxed and closed.
Imagine smiling more inwards than outwards.

Face massage I
Place the fingertips of your right hand on your forehead just above your left eyebrow.
Place the fingertips of your left hand directly above them.
Stroke your forehead slightly outwards towards your hairline with your left hand.
Hold your right hand gently against this, so that the skin stretches slightly.
Return your left hand by gently gliding your fingernails over the skin.
Massage three times.
Now switch to the right side of your forehead and massage three times there as well.

Face massage II
Place the fingertips of both hands side by side in the centre of your forehead.
Stroke your forehead down to your temples.
Apply pressure here with a gentle circular motion.
Return your hands to the centre.
Massage three times.

Face massage III
Start at the bridge of your nose and stroke along your cheekbones to your temples.
Continue from the bridge of your nose, under your cheekbones, to your temples.
Finally, from your chin, following your jawline to your temples.

Massage each area three times in succession finishing with a circular motion at the temples.

Brow massage I

Grasp your left eyebrow at its highest point between the thumb and forefinger of your left hand. Make sure you also grasp the muscle running along the brow. Grasp the brow immediately to the right of this point with the thumb and forefinger of your right hand.

Gently pull the brow away from your face and move your fingers up and down three times in opposition to each other. You should feel a distinct but not painful stretching sensation.

Reposition your grip about a finger's width to the right each time and massage in this way until you reach the highest point of your right eyebrow.

Now grasp the skin between your eyebrows and above the bridge of your nose vertically and massage it by moving both hands three times in opposition to each other, to the right and left.

Repeat the massage a total of three times.

Brow massage II

Place your thumbs under the inner corners of your orbits, at the level of your eyebrows, applying comfortable pressure.

Now, grasp the skin and muscles from above with your index and middle fingers and massage firmly between your fingers. Knead and smooth the skin with circular motions, pulling it slightly away from your face.

Move your fingers a little further out, to where the edge of the orbit forms a small step, and massage this area as well, kneading and smoothing.

Repeat the massage three times in each position.

Brow massage III

Using your index and middle fingers, firmly stroke your eyebrows from the centre outwards, smoothing and tightening the skin between them. Support this by firmly tensing your forehead muscles.

Place the tip of your left index finger slightly below the centre of your forehead and gently stretch the skin upwards. Place the tip of your right index at the bridge of your nose. Now firmly stroke the area between your eyebrows with your right index upwards towards the left index.

Repeat this movement a total of three times.

Chin massage I

Grasp the skin and muscle of your chin below the left corner of your mouth with the thumb and forefinger of your left hand, and immediately to the right of that with the thumb and forefinger of your right hand.

Gently pull your chin away from your face and move your fingers up and down three times in opposition to each other. You should feel a distinct, but not painful, stretching sensation.

Reposition your grip about a finger's width to the right each time and massage your chin down to just below the right corner of your mouth.

Now, grasp your chin vertically at the level of the left corner of your mouth and massage by moving both hands three times in opposition to each other, to the right and left. Repeat this in the middle of your chin and at the level of the right corner of your mouth.

Repeat the massage three times.

Chin massage II

Place your thumbs at the corners of your mouth, under the edge of your lower jaw. Now, using your index and middle fingers, grasp the skin and muscles around the corners of your mouth and chin from above and massage them firmly between your fingers. Knead and smooth the skin with circular motions, gently pulling it away from your face.
Move your hand to the centre of your chin and massage accordingly.
Repeat the massage three times in each position.

Chin massage III
Place the tips of your index fingers on your chin below the corners of your mouth, applying light pressure, and gently stroke the corners of your mouth outwards and upwards.
Apply pressure to a point below your cheekbones using a light circular motion.
Repeat the exercise three times.

“Broad grin”
Pull the corners of your mouth to the side with all your force, as far as you can.
At the same time, pull your cheeks upwards as hard as you can, as high as possible.
Hold them there for about 20 seconds. Holding the tension should be strenuous and lead to a feeling of fatigue or "sourness" in the muscles of your cheeks.
Now relax slowly and consciously.

“Pull-up”
Open your mouth about halfway.
Roll your lips over your teeth.

Now, place your index fingers in an arc on your cheekbones with firm pressure and press your cheeks firmly downwards.
Now, raise your cheeks again against the resistance of your lips and fingers. To do this, pull the corners of your mouth firmly upwards and outwards. Also engage the muscles in the corners of your eyes.
It should be quite strenuous to pull your cheeks up against the resistance.
Avoid frowning.
Hold the tension for a moment until you notice that your muscles begin to feel "sour."
Release the tension slowly.
Do ten pull-ups in a row.

“Mask”
Open your mouth slightly.
With firm pressure, place your index fingers from the corners of your eyes across your cheekbones and your middle fingers along the creases that run from your nostrils to the corners of your mouth.
Tighten the skin of your cheeks by spreading your fingers and pulling your hands down and outwards.
Against the resistance of your fingers, lift the corners of your mouth and pull your cheeks together.
Really exert yourself and build up as much tension as possible.
Release the tension slowly.
Do ten contractions in a row.

Conclusion
Gently and evenly tap your entire face, except for your eyes, with your fingertips.
Tap for about half a minute to a minute.
Pay special attention to the area around your eyebrows and the area below your mouth.

Finally, gently stroke your face three times with the palms of your hands in one sweeping motion, from your forehead over your cheeks to your temples.

These descriptions only serve to comprehend the interventions in the trial. They don't replace the full illustrated instructions, which will be published separately as a therapeutic manual and/or application software.

Supplementary Data 2

Translation of the items of the **Fragebogen zu Leistungsfähigkeit und Wohlbefinden** (questionnaire for capability and wellbeing) by the Swiss Group Project "Depression: Complete Remission/Well-Being (Bondolfi G., Aubry J.M., Baciu C., Calanchini C., Hatzinger M., Hemmeter U., Holsboer-Trachsler E., Seifritz E., 2010).

During the last week including day of assessment:

1) Do you have the impression to have regained your accustomed level of capability and wellbeing? yes/no
2) How do you rate your current condition? 1-100%

**Professional life**	
I have regained my complete, accustomed capability	not agreed/agreed 1-10
I am optimistic, confident, and full of energy	not agreed/agreed 1-10
I bear my responsibility	not agreed/agreed 1-10
**Social life/relationships**	
I have regained my complete, accustomed capability	not agreed/agreed 1-10
I feel happy most of the time	not agreed/agreed 1-10
I take part in activities and have fun doing so	not agreed/agreed 1-10
**Family life**	
I have regained my complete, accustomed capability	not agreed/agreed 1-10
I feel happy most of the time	not agreed/agreed 1-10
I take part in activities and have fun doing so	not agreed/agreed 1-10

2 Supplementary Figures and Tables

2.1 Supplementary Figures

Overview of study procedure and assessments

	Screening	Study period				
Visit	0	1	2	3	4	5
Time (in weeks)		0	3	6	9	12
Study information and informed consent	x					
Inclusion and exclusion criteria	x					
Demografic and clinical data	x					
Randomization		x				
Instruction FareWell Depression (including a physiotherapeutic supervision one week later)		IG		CG		
Instruction control exercise (relaxation)		CG				
Administration of the FaReWell Depression program		IG	IG	IG	IG	IG
				CG	CG	CG
Administration of the control exercise		CG	CG	CG		
MDBF pre/post exercise (every two weeks)		x	x	x	x	x
MADRS		x	x	x	x	x
PHQ-9		x		x		x
SHAPS-D		x		x		x
Questionnaire assessing well-being and functioning		x		x		x
Standardized portrait photos		x		x		x
Assessment of tension and discomfort		x		x		x
Assessment of adverse effects			x	x	x	x

Supplementary Figure 1. Study Schedule
Gray-shaded areas indicate visit-independent and self-administered completion of the exercises as well as the self-rating (MDBF). Abbreviations: IG = Intervention Group, CG = Control Group, MDBF = Multidimensional Mood State Questionnaire, MADRS = Montgomery–Åsberg Depression ating Scale, PHQ-9 = Patient Health Questionnaire–9, SHAPS-D = Snaith–Hamilton Pleasure Scale.

2.2 Supplementary Tables

Supplementary Table 1 Demographic and clinical characteristics of the dropouts and study sample

	**Dropouts** (n=9)	**Study Group (IG & CG)** (n = 36)	
		Mean (s.d.)	*p*-Value
Sociodemographic characteristics			
Age (years)	35.67 (12.87)	43.00 (12.67)	.130[1]
Sex (female/male)	7/2	26/10	.736[2]
Years of Education	15.88 (1.76)	14.26 (2.24) (n=25)	**.049[1]**

Clinical characteristics			
Number of depressive episodes	3.50 (2.34) (n=6)	3.91 (3.75) (n=33)	.799[1]
Number of suicide attempts	0.00 (0.00)	0.139 (0.42)	.336[1]
Lifetime psychiatric comorbidity (yes/no)	2/7	12/24	.520[2]
Anxiety disorders	1	6	-
Eating disorders	1	2	-
ADHD	0	2	-
Others	0	2	-
Psychotherapeutic Treatment (yes/no)	9/0	30/6	.188[2]
Medical treatment at baseline (yes/no)	3/6	20/16	.233[2]
Mono therapy	3	16	-
Combined therapy	0	3	-
Augmentation therapy	0	1	-

Notes: Significant group differences are highlighted in ***bold.*** [1]*one-wayANOVA,,* [2]*Chi-squared t-test. Abbreviations: IG = Intervention Group, CG = Control Group, ADHD = Attention-Deficit-Hyperactivity Disorder.*

Supplementary Table 2 Facial sensations and adverse effects

	**Intervention Group (IG)** (n = 17)	**Control Group (CG)** (n = 19)
	Mean (s.d.)	Mean (s.d.)
**Tension and paresthesia**		
week 0 (yes/no)	5/12	6/13
Location (Mouth/Eyes/Other)	0/4/1	5/1/0
week 6 (yes/no)	4/13	3/16
Location (Mouth/Eyes/Other)	0/4/4	2/1/3
week 12 (yes/no)	2/12	4/13
Location (Mouth/Eyes/Other)	1/0/1	2/1/1
**Adverse Effects**		
week 3 (yes/no)	5/12	3/15

Location (Skin/Pain/Psych. Burden/Other)	2/3/0/0	0/0/3/0
week 6 (yes/no)	5/12	3/16
Location (Skin/Pain/Psych. Burden/Other)	2/1/0/2	0/0/3/0
week 9 (yes/no)	1/13	4/14
Location (Skin/Pain/Psych. Burden/Other)	0/1/0/0	0/2/1/1
week 12 (yes/no)	2/12	5/12
Location (Skin/Pain/Psych. Burden/Other)	0/1/0/1	1/3/0/0
